# The Role of Pre-surgery Clinical Communication on Metabolic and Bariatric Surgery Outcomes: A Prospective Study

**DOI:** 10.1007/s11695-025-07772-1

**Published:** 2025-03-13

**Authors:** Ana João Ferreira, Irene P. Carvalho

**Affiliations:** 1 Faculty of Medicine, University of Porto - Alameda Professor Hernâni Monteiro, 4200-319 Porto, Portugal; 2 Department of Clinical Neurosciences and Mental Health, Faculty of Medicine, University of Porto - Alameda Professor Hernâni Monteiro, 4200-319 Porto, Portugal; 3 CINTESIS@RISE, Faculty of Medicine, University of Porto - Alameda Professor Hernâni Monteiro, 4200-319 Porto, Portugal

**Keywords:** Communication Assessment Tool, Metabolic and bariatric surgery, Doctor-patient relationship, Patient clinical outcomes

## Abstract

**Background:**

Research shows that a positive doctor-patient relationship plays an important role in patient outcomes. However, the influence of their communication during the pre-surgery preparatory consultation (PC) for metabolic and bariatric surgery (MBS) remains unclear. The goal of this study was to inspect the association between patients’ perceptions of doctor-patient communication (DPC) in the PC for MBS and the results of the MBS.

**Methods:**

This prospective cross-sectional study included 89 adult patients undergoing MBS at a hospital. Before the surgery, patients’ perspectives regarding DPC were assessed with the Communication Assessment Tool (CAT). One month after the surgery, participants’ levels of well-being were assessed through the 36-Item Short Form Survey (SF-36). Other clinical data were obtained through patients’ electronic records. Data were analyzed with regression models.

**Results:**

In the adjusted models, associations with the quality of doctor-patient communication (*p* < 0.05) were found for the following outcomes: weight loss, body mass index decrease, and patient well-being regarding bodily pain and social functioning. Significant differences (*p* < 0.05) were also found for digestive complaints and for patient perception of physician post-surgery support.

**Conclusions:**

DPC in the preparatory consultation has a positive effect on the clinical results of MBS. More studies are necessary for inspection of the generalizability of these findings.

**Graphical Abstract:**

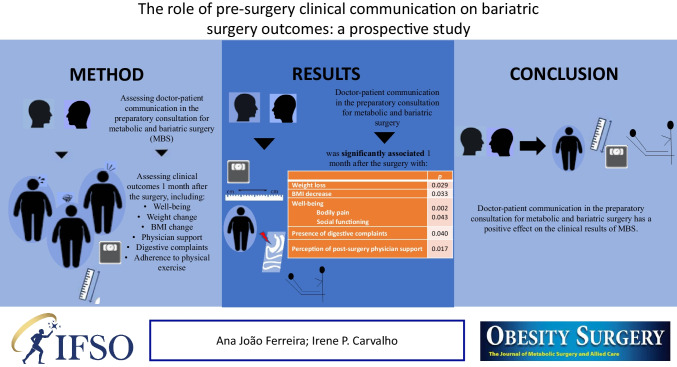

## Introduction

Although there are several types of therapies for the treatment of obesity, metabolic and bariatric surgery (MBS) often shows superior effectiveness, when compared to non-surgical treatment options, with a greater probability of decreasing other comorbidities [1–3]. MBS has been associated with positive effects on lipid profile, cardiovascular risk, type 2 diabetes, arterial hypertension, dyslipidemia and quality of life, among other aspects [1, 4–7].

Despite these positive results, many patients with obesity do not undergo MBS lightly but rather consider choosing more conservative treatment options because of significant fear of surgery and its possible side effects [8]. Also, maintaining or increasing weight loss during the first year after MBS depends on the information that patients possess and, on their expectations, in addition to proper nutritional habits, regular physical activity, and the interplay of genetics, neuro-hormonal factors and other individual differences [4, 9–12]. The preparatory appointment for MBS can assume an important role in this context, and the quality of doctor-patient communication (DPC) in the preparatory appointment for MBS might influence patients’ outcomes following the surgery.

Positive effects of patient-centered communication have been documented in various studies of different areas of medicine, and the focus on patient-centered communication is widely supported as a key component in the provision of secure and effective health care and continuous improvement of health outcomes [13–15]. For example, the term “patient activation” refers to the knowledge, skills, and confidence required for a patient to understand their role in their process of health care and feel capable of accomplishing it. Previous research indicates that patient outcomes tend to be comparatively better when patients understand the information and actively participate in their health process [14]. Furthermore, a stronger patient-doctor relationship, i.e., the presence of a therapeutic alliance and patient-focused communication, could lead to more favorable clinic results [10, 11, 14–18]. People with obesity might not have the confidence to manage their health, but they consider communication with the doctor as a key ingredient for self-care management [17].

Studies in MBS have assessed doctor-patient relationship in general. In sum, they indicate that a positive relationship, along with other predictors such as weight loss before surgery, among others, are associated with patients’ outcomes, namely, with a greater likelihood of adherence to follow-up care and reduction of post-surgical complications [4, 19, 20]. However, the underutilization of metabolic and bariatric surgery, despite its efficacy and safety, remains a significant issue, driven by factors such as health insurance barriers, weight stigma, and suboptimal patient-provider communication. Shared decision-making (SDM) has been proposed as a strategy to address these challenges, as it promotes effective communication and patient-centered care [21, 22]. While these factors have been well documented, the specific influence of doctor-patient communication during the pre-surgery preparatory consultation (PC) on MBS outcomes remains unclear. Bridging this gap, the goal of this study was to inspect the association between patients’ perceptions of doctor-patient communication in the preparatory consultation for MBS and the results of the surgery. In previous research, MBS has been associated with decreased weight and body mass index, and with improved quality of life [23, 24]. The MBS outcomes may be influenced by several factors, including doctor-patient communication in the preparatory consultation.

## Methods

### Participants

In this prospective cross-sectional study, participants were all the patients who underwent MBS at the Integrated Obesity Responsibility Center of the São João Hospital and University Center, Portugal, between October of 2023 and January of 2024. The inclusion criterion was being 18 years or older. The exclusion criterion was the presence of any neurological or cognitive disability that compromised participation, as identified in patients’ clinical records or detected during the interaction with the patient.

### Measures

#### Socio-demographic and Clinical Characteristics

All baseline characteristics, including pre-surgical weight, and pre-surgery BMI, were retrieved from the participants’ hospital electronic records. Research has suggested that patients’ satisfaction ratings can be influenced by their anxiety levels [25]; thus, patients’ levels of anxiety in the preparatory session for MBS were assessed with the State-Trait Anxiety Inventory Form Y (STAI-Y) [26–31].

#### Doctor-Patient Communication in the Preparatory Consultation for Surgery

Patients’ perspectives regarding their physician’s communication in the MBS preparatory consultation were assessed with the Communication Assessment Tool (CAT). Overall CAT scores range from 14 (minimum) to 70 (maximum). Higher scores indicate greater competence in health communication behavior [32–34]. The CAT has shown good reliability and validity properties across studies [34–36].

### Outcomes

Participants’ well-being after the MBS was assessed with the 36-Item Short Form Survey (SF-36) [37]. The SF-36 questionnaire is a self-report instrument, thus allowing for the possibility of assessing the patient’s subjective sense of quality of life. It comprises 8 dimensions that can be converted into two major components: a physical component (physical functioning, physical performance, bodily pain, and general health) and a mental component (vitality, mental health, social functioning, and emotional performance). The resulting scores indicate levels of quality of life, ranging from 0 (worst health status) to 100 (best health status). Good reliability properties have been reported for the Portuguese version used in this study [38–40].

Post-surgery clinical outcomes collected from patients’ electronic records included post-surgery weight, post-surgical BMI, and adherence to the therapeutic plan, which consisted of physical activity (initiating any light walking). In addition, patients were asked about the presence of digestive complaints (e.g., vomiting, nausea, constipation, abdominal discomfort) with response options of 0 (no) or 1 (yes).

Participants additionally rated a question on the quality of the physician’s support/response during the post-surgery follow-up period as either 0 (poor) or 1 (good). The post-surgery follow-up period consisted of the first month after surgery, including the hospitalization period (lasting about two/three days, on average).

### Procedures

**Fig. 1:**
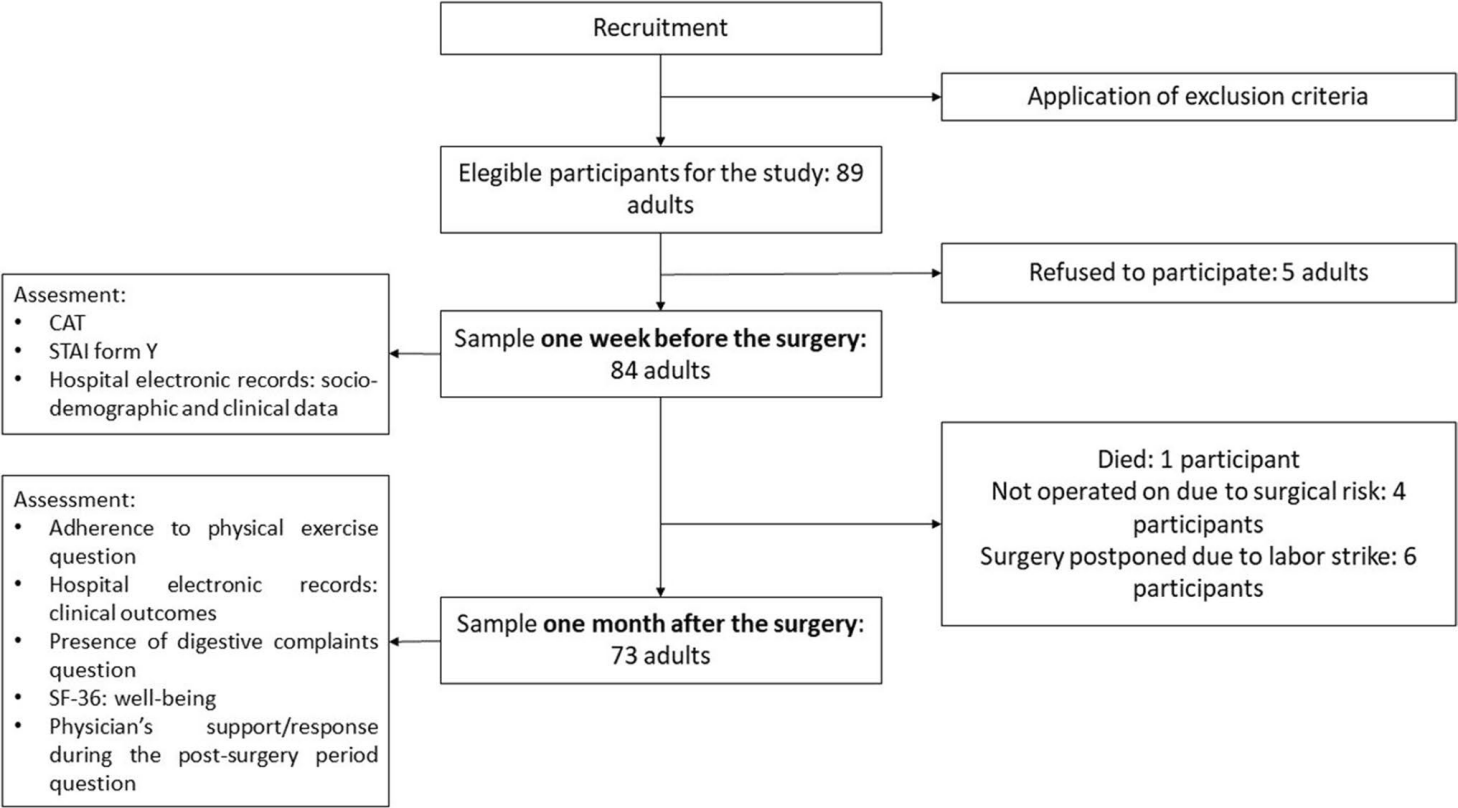
Study design

Patients were invited to the study and responded to the questionnaires via telephone call. One week before the surgery, their perspectives were collected regarding doctor-patient communication in the preparatory consultation for MBS. This PC is typically conducted between the surgeon and the patient about all steps related to the surgery. Patients’ anxiety levels during that PC were also assessed at this point. The clinical results of the surgery were evaluated one month after the MBS. Research shows that significant changes in patient outcomes, such as weight loss and metabolic markers, can become evident within the first month after MBS [41]. Evaluating these factors at this time provides valuable insights into the early effectiveness of the surgery and its impact on the patient’s health. The study’s design and procedures are shown in Fig. 1.

#### Statistical Analyses

Data were analyzed in SPSS®, version 29, and were expressed as absolute and relative frequencies, means and standard deviations, or medians and interquartile ranges, as appropriate. Logistic regression was used to assess the influence of DPC in the MBS preparatory encounter on patient adherence to the physical activity plan. Linear regression was used to evaluate the association of doctor-patient communication, respectively, with weight change, BMI change, and patient well-being (on the SF-36 dimensions) 1 month after the surgery. Weight change and BMI change were calculated, respectively, by the difference between post-surgical weight/BMI and pre-surgical weight/BMI, with negative values indicating a reduction in weight/BMI compared to prior to surgery. The models were adjusted for factors that could influence the outcomes, namely, patient anxiety at the time of the preparatory appointment for the MBS [26, 27, 42], age, gender, history of previous surgeries for obesity, and family history of obesity [43]. The data were confirmed to be appropriate for regression models. Only the distributions for the presence of digestive complaints and for physician support/response after the surgery, respectively, posed problems to the assumptions of the logistic regression [44], and the Mann–Whitney test was used instead. A *p*-value below 0.05 was considered statistically significant.

## Results

### Baseline Characteristics

Of the initial 89 eligible patients contacted during the period of recruitment, 84 accepted to participate in the study (Fig. 1). Most were women (77.4%) and mean age was about 48 years old (*SD* = 11.60). Clinically severe obesity (class III obesity) was the most common type of obesity in the sample (67.9%). The sample’s characteristics are presented in Table 1.

**Table 1:** Sample’s characteristics at baseline

		Participants (*N* = 84)
Age (years)		
	Mean (*SD*)	47.76 (11.60)
Gender - *n* (%)		
	Women	65 (77.4%)
	Men	19 (22.6%)
Household composition - *n* (%)		
	Living alone	10 (11.9%)
	Living with family	74 (88.1%)
Working status *- n* (%)		
	Employed	48 (57.1%)
	Unemployed/retired/housekeeper	36 (42.9%)
Type of surgery *- n* (%)		
	LAGB removal or RYBG reversion	14 (16.7%)
	RYBG or SG or BPD/DS	70 (83.3%)
Reason for surgery/onset of obesity *- n* (%)		
	Obesity since childhood	21 (25.0%)
	Obesity during adulthood	16 (19.0%)
	Obesity after pregnancy	13 (15.5%)
	Nutritional problems or reflux	6 (7.1%)
	Weight regain or reflux symptoms after metabolic and bariatric surgery	28 (33.3%)
Previous metabolic and bariatric surgery - *n* (%)		
	Yes No	37 (44.0%) 47 (56.0%)
Family history of obesity - *n* (%)		
	Yes No	57 (67.9%) 27 (32.1%)
Height (meters)		
	Mean (*SD*)	1.64 (0.09)
Pre-surgical weight		
	Mean (*SD*)	114.74 (23.32)
Pre-surgical BMI (m/kg^2^)		
	Mean (*SD*)	42.78 (7.82)
BMI classification *- n* (%)		
	Normal range	2 (2.4%)
	Overweight	1 (1.2%)
	Class I obesity - obesity	7 (8.3%)
	Class II obesity - severe obesity	17 (20.2%)
	Class III obesity - clinically severe obesity	57 (67.9%)
State anxiety (STAI Form Y-1) ^a^		
	Mean (*SD*)	38.53 (13.59)
Trait anxiety (STAI Form Y-2) ^a^		
	Mean (*SD*)	38.42 (10.67)

*SD* standard deviation, *LAGB* laparoscopic adjustable gastric band, *RYBG* Roux-en-Y gastric bypass, *SG* sleeve gastric, *BPD/DS* Biliopancreatic diversion with duodenal switch, *BMI* body mass index, *Class I-III obesity* obesity is subdivided into categories based on body mass index (BMI)

^a^*N* = 77 participants due to missing responses. The State-Trait Anxiety Inventory Form Y (STAI-Y) scores range from 20 (minimum possible value) to 80 (maximum possible value)

### Doctor-Patient Communication in the Preparatory Consultation

Mean quality of the DPC, as rated by the participants on the CAT, was 64.67 out of a possible maximum of 70 (*SD* = 7.98), with values ranging from 24 to 70. Of the questions addressed in the CAT, the one with the highest mean was “treated me with respect” (*M* = 4.89, *SD* = 0.35) and the second was “talked in terms I could understand” (*M* = 4.87, *SD* = 0.37). The questions rated the lowest by the patients were “discussed next steps, including any follow-up plans” (*M* = 4.13, *SD* = 1.29), “encouraged me to ask questions” (*M* = 4.42, *SD* = 0.91) and “involved me in decisions as much as I wanted” (*M* = 4.42, *SD* = 0.99).

### Post-surgery Outcomes

Of the 84 initial participants, 73 completed the post-surgery phase (87% retention rate; Fig. 1). Table 2 shows that 57.5% of the sample failed to adhere to the prescribed physical activity plan (i.e., initiating any light walking after the surgery). Mean weight change one month after the MBS was 9.19 kg (*SD* = 6.48), with the difference between pre-surgical and post-surgical weight varying between losing 20.10 kg and gaining 15 kg. Mean BMI decrease between before and 1 month after the surgery was − 3.46 kg/m^2^ (*SD* = 3.05), ranging from a BMI decrease of 17.29 kg/m^2^ to an increase of 3.20 kg/m^2^. For both these outcomes, additional analyses showed no significant differences between participants who adhered and who did not adhere to the physical activity therapeutic plan (weight change: *t*(63) = 1.083; *p* = 0.141; BMI change: *t*(63) = 1.616; *p* = 0.111). One month after the MBS, patients reported the highest well-being levels for the dimensions of social functioning and of bodily pain, whereas emotional performance and vitality were the dimensions with the lowest means. Most participants presented no digestive complaints after the surgery (79.5%). Most rated the doctor’s support/response in the follow-up period after the surgery, as “good” (83.6%).

**Table 2:** Outcomes: Therapeutic adherence, clinical results, and perception of physician support one month after metabolic and the bariatric surgery

		Participants (*N* = 73) ^a^
Adherence to the physical activity plan - *n* (%)		
	Yes No	31 (42.5%) 42 (57.5%)
Difference between pre-surgical and post-surgical weight		
	Mean (*SD*)	–9.19 (6.48)
Difference between pre-surgical and post-surgical BMI		
	Mean (*SD*)	–3.46 (3.05)
Patient well-being (on the SF-36)		**Participants (** ***N*** **= 72)** ^**b**^
Physical functioning	Mean (*SD*)	62.92 (25.31)
Physical performance	Mean (*SD*)	61.46 (39.79)
Emotional performance	Mean (*SD*)	32.41 (40.72)
Bodily pain	Mean (*SD*)	73.78 (29.23)
General health	Mean (*SD*)	67.22 (21.61)
Vitality	Mean (*SD*)	57.12 (23.39)
Social functioning	Mean (*SD*)	79.91 (27.33)
Mental health	Mean (*SD*)	74.03 (23.61)
Physical component	Mean (*SD*)	66.35 (15.12)
Emotional component	Mean (*SD*)	60.12 (14.72)
		**Participants (** ***N*** **= 73)** ^**a**^
Presence of digestive complaints - *n* (%)		
	Yes	15 (20.5%)
	No	58 (79.5%)
Patient perception of physician support/response after surgery *- n* (%)		
	Good	61 (83.6%)
	Poor	11 (15.0%)
	No answer	1 (1.4%)

*SD* Standard deviation, *BMI* body mass index; negative values mean that the weight or the BMI decreased one month after metabolic and the bariatric surgery

^a ^*N* = 73 because this was the final number of eligible participants who underwent surgery and who were followed up after one month. ^b^*N* = 72 due to one missing response

### Effects of Communication in the Preparatory Consultation for MBS on the Outcomes

Table 3 shows that a non-significant effect was found between doctor-patient communication in the preparatory consultation for MBS and post-MBS adherence to physical activity (*p* = 0.770)*.* The effects were otherwise significant for all other outcomes 1 month after the surgery, and preliminary analyses further showed that pre-surgery weight and BMI at baseline were both uncorrelated with communication (*r*_s_ = 0.007;* p* = 0.956; *r*_s_ = 0.135;* p* = 0.221, respectively). Specifically, the greater the patient’s perceived quality of communication with the physician in the PC for MBS, the greater the post-surgery weight loss (*p* = 0.029), BMI decrease (*p* = 0.033), well-being bodily pain (*p* = 0.002), social functioning (*p* = 0.043) and total mental component (*p* = 0.012), and perception of physician support/response in the post-surgery follow-up period (*p* = 0.017). Only the association between communication and digestive complaints had the opposite direction, that is, the greater the quality of the communication, the more the patients reported having digestive complaints after the surgery (*p* = 0.040).

**Table 3:** Effects of communication on the outcomes

OUTCOMES		*β*	Standard error	*p-*value^a^	CI	*η* ^2^
Adherence to the physical activity plan		0.012	0.041	0.770	0.934–1.097	
Weight		-0.151	0.67	**0.029**	-0.285 – (-0.016)	0.076
BMI		-0.091	0.042	**0.033**	- 0.175 – (-0.007)	0.072
Well-being: **Physical component**		0.395	0.199	0.052	-0.004–0.794	0.061
	Physical functioning	0.269	0.331	0.420	-0.393–0.931	0.011
	Physical performance	-0.482	0.601	0.426	-1.686–0.721	0.011
	Bodily pain	1.305	0.395	**0.002**	0.514–2.096	0.154
	General health	0.489	0.320	0.132	-0.152–1.129	0.037
Well-being: **Emotional component**		0.522	0.201	**0.012**	0.121–0.923	0.101
	Emotional performance	0.598	0.581	0.308	-0.565–1.761	0.017
	Vitality	0.344	0.318	0.283	-0.292–0.980	0.019
	Social functioning	0.781	0.377	**0.043**	0.026–1.536	0.067
	Mental health	0.364	0.309	0.244	-0.255–0.983	0.023
		**Mann-Whitney** ***U***		*p-*value^b^		
Presence of digestive complaints						
	0- No (*n*=58; *Mdn*_CAT_=66.00; *IQR*=60–70) 1- Yes (*n*=15; *Mdn*_CAT_=70.00; *IQR*=58–70)	288.000		**0.040**		
Physician support/response after surgery						
	0- Poor (*n*=11; *Mdn*_CAT_=60.00; *IQR*=58–66) 1- Good (*n*=61; *Mdn*_CAT_=68.00; *IQR*=63–70)	186.500		**0.017**		

*BMI* body mass index, *CI* confidence interval, *Mdn* median, *IQR* interquartile range, *CAT* Communication Assessment Tool, *M* mean, *SD* standard deviation

^a^Model adjusted for patients’ state anxiety, age, gender, family history of obesity, and previous metabolic and bariatric surgery; ^b^ Mann-Whitney *U* test due to imbalanced groups

## Discussion

The findings suggest that the quality of doctor-patient communication in the preparatory consultation for MBS has a positive effect on MBS outcomes, namely, on post-surgery loss of weight and BMI, less bodily pain, better social function, and greater perceived physician follow-up support. These results are suggestive of the importance of doctor-patient communication in the preparatory consultation for bariatric surgery. They are in line with previous research that emphasizes the positive impact of interventions aimed at improving the patient-professional relationship on the health of the populations in various areas of medicine [19, 26, 27], and extend previous MBS research focusing on the quality of doctor-patient relationship in general to the specific context of communication in the PC for MBS. The findings also resonate with studies identifying barriers to effective shared decision-making (SDM) in MBS, such as limited patient awareness and suboptimal communication, which are crucial for enhancing patient outcomes. The decreases in weight and BMI observed 1 month after the surgery in the sample taken as a whole are generally in line with those reported in other samples [45], and improved quality of life has also been associated with MBS in previous research [23, 24]. By addressing these communication barriers, particularly during the PC, it may be possible to improve patient engagement and ultimately the success of MBS interventions [21, 22].

These findings contribute to the growing body of evidence supporting the efficacy of doctor-patient communication, namely, regarding its potential to enhance patient experiences and mitigate healthcare costs. For example, the association found between improved communication and bodily pain suggests a potential way for cost reduction in future healthcare expenditures, namely, due to decreased need for further services resulting from fewer side effects and greater treatment satisfaction [14–16].

Adherence to physical activity was the only outcome on which DPC had a statistically non-significant effect. In MBS, physical exercise is often indicated as part of the therapeutic plan, although a meta-analysis showed only a modest effect of post-surgical exercise on additional weight loss and fat loss [46]. In this study, more than half of the patients undergoing MBS did not perform any physical activity perhaps because, just one month after the MBS, the indications given are mainly that patients avoid heavy exertion due to the need for healing. A longer follow-up period could provide a more accurate picture about the role of communication in the preparatory encounter for MBS on patients’ post-surgery adherence to physical activity.

In addition, the significant association observed between pre-surgery communication and patients’ digestive complaints after the surgery contrasted with the direction of the associations for all the other significant outcomes in this study. This finding suggests that increased quality of DPC before the surgery could contribute to patients’ greater comfort to report on their complaints and disclosing of more information after the surgery [47]. This result was obtained with the Mann–Whitney test, due to the imbalanced groups, and other aspects could be interfering in the association, for which the test was unadjusted. Nevertheless, the same test was applied, for the same reasons, to the post-surgery support received from the surgeon and the results followed the expected direction in this latter case. Both these aspects (digestive complaints and post-surgery support received from the surgeon), as well as exercise adherence, were assessed on a dichotomous (either 0 or 1) response option, and future studies based on Likert-type scales can cast further light onto these results by various levels.

Regarding the outcomes of the patients’ reports about the DPC on the CAT questionnaire, the results in this sample were similar to those identified in another research [32, 35]. Given the importance that doctor-patient communication can have on patient outcomes, as observed in this study, this finding underscores the necessity for targeted training initiatives catering to healthcare professionals, particularly in domains such as encouraging patients to ask questions, involving patients in shared decision-making processes, and equipping patients with a comprehensive understanding of their clinical journey (tailored to their needs). These findings illuminate a consistent need, within the healthcare sector, for tailored training programs to address the identified gaps [32, 34].

This study had some limitations. First, the relatively small sample size could prevent the emergence of further significant results. Also, the study was conducted in one center, which, even though consisting of one of the largest hospitals in the country, limits the generalization of the results. Future studies with larger samples, including in different hospitals across the country, with a longer post-surgery follow-up period, could shed further light on these results. Second, in the pre-surgery period, the CAT and the STAI-Y (assessing anxiety) were not applied immediately after the doctor’s appointment, but later via telephone call, which could introduce some memory bias. Third, it could be possible that demand characteristics in this sample might influence these results. For example, some patients had obesity since childhood, whereas others underwent surgery for obesity after pregnancy, or for nutritional problems or reflux. The models were adjusted for potential confounders, although it is possible that other variables that were not considered could influence the results.

Despite these limitations, the prospective character of this study, which involved the assessment of communication in the PC before the surgery, and the assessment of the outcomes after the surgery, along with the inclusion of both patients’ perceptions and objective measures, strengthens the study’s results. Future studies are needed, beyond 1 month after the surgery, for the continuous monitoring of the patients’ health changes associated with the quality of DPC. In the future, identifying the best methods of communication to apply in the PC for MBS could also help in training physicians.

## Conclusion

This study showed a positive effect of doctor-patient communication in the PC on the results of metabolic and bariatric surgery, not only regarding loss of weight and decreased BMI one month after the surgery, but also in terms of both emotional and physical well-being, and perceived surgeon support after the surgery. These results are indicative of the importance of doctor-patient communication in the specific context of the preparatory consultation for MBS. Further studies in this context may contribute to improving clinical practice in the future.

## Data Availability

The data that supports the findings of this study are available from the corresponding author upon reasonable request.
